# Mock Jurors’ Determinations of Guilt in Sexual Offences Involving Male Victims: A Systematic Review

**DOI:** 10.1177/15248380251366264

**Published:** 2025-09-16

**Authors:** Madeleine Millar, Siobhan Weare, Dominic Willmott

**Affiliations:** 1 Lancaster University, UK; 2 Loughborough University, UK; 3 SWPS University, Wroclaw, Poland

**Keywords:** jury decision-making, sexual violence, gender, male victims, rape myths, vulnerability

## Abstract

Research examining perceptions of male victims of sexual violence has been neglected, particularly regarding jury attitudes toward these victims. It is important to recognize whether jurors, as community decision-makers, hold falsehoods, stereotypes, and gendered beliefs about male victims of sexual violence and whether these beliefs affect their legal attitudes and verdict decisions. A systematic review of peer-reviewed, experimental studies published between 1980 and 2024 was conducted. A total of 11 databases, alongside ascendancy, descendancy, and gray literature searches across two additional platforms, were searched using 18 search strings, yielding 21 sources which met the pre-registered inclusion criteria. Reviewers assessed all sources in terms of quality using two separate measures. Synthesis of the reviewed sources suggests that mock jurors are typically less punitive toward defendants in sexual violence cases with male victims, compared to those with female victims. While half of the sources find no effect of victim gender on mock jurors’ verdict decisions, this is accompanied by findings indicating that mock jurors hold harsher attitudes and beliefs toward male victims. Several sources also suggest that male victims’ ethnicity, sexuality, and gender identity are additional characteristics that affect mock jury attitudes and legal outcomes. Mock jurors were typically more lenient toward alleged perpetrators of sexual violence and held harsher and more punitive attitudes toward Black, gay, or transgender male victims in sexual violence cases. Finally, we discuss implications for policy and practice, and recommendations for future research.

One in 21 men report being raped or sexually assaulted since the age of 16 (Office for National Statistics [ONS], 2020). However, the prevalence of male sexual violence may be higher than these figures suggest. In 2021, research commissioned by the charity Mankind UK found that 1 in 7 men in the United Kingdom report being coerced into sex and 1 in 10 men report experiences of rape or nonconsensual penetration (Savanta ComRes, 2021). Stigma toward male victims of sexual violence can function as a significant barrier for men to make informal disclosures and/or formally report their assault (Weare et al., 2024); for example, 4 in 5 men do not report their rape or assault by penetration to the police (ONS, 2021). Thus, perceptions of male victims of sexual violence may have tangible effects on their access to justice. However, despite the prevalence of reported and unreported male sexual victimization, research examining perceptions of male victims^1^ in sexual violence cases has been severely neglected (Davies et al., 2008).

Understanding lay perceptions of male victims of sexual violence is important, especially in the context of jury decision-making, where jurors, as community decision-makers, may perpetuate stigmatizing attitudes about and toward male victims of rape and sexual assault, and where juror decisions represent community sentiment toward social issues. Where jurors contribute to errors of justice, this can have extensive consequences (e.g., psychological harm), not only for those who have suffered a potential miscarriage of justice, but also in terms of public trust and confidence in the ability of the criminal legal system to administer justice (Naughton, 2007; Quirk, 2007).

It is critical that jury verdicts reflect decision-making based on the strength of evidence rather than prejudicial attitudes. However, research suggests that attitudes toward male victims of sexual violence are heavily influenced by false assumptions about how men, and victims of sexual violence more generally, should act—also known as rape myths (Ayala et al., 2018; Struckman-Johnson & Struckman-Johnson, 1992). This includes beliefs about whether a man can be raped by a woman (e.g., “I would have a hard time believing a man who told me that he was raped by a woman”) and expectations surrounding how male victims should behave before, during, and after their assault (e.g., “Any healthy man can successfully resist a rapist if he really wants to”) (Melanson, 1998). Importantly, these myths extend to legal decisions in actual criminal cases. For instance, in *R. v. Armstrong*, where a defendant was charged with forcible sodomy, the trial judge instructed the jury to acquit on the basis that there was “‘not sufficient evidence’ of the complainant’s non-consent” as the complainant reportedly had an erection during the assault (cited in Morgan-Taylor & Rumney, 2004).

Given calls for “systematic research” on male sexual assault (Smith et al., 1988, p. 112), and “assessments of blame towards male victims” of sexual violence more specifically (Davies et al., 2001, p. 608), we aim to understand how jurors make decisions and assign verdicts in cases where men are victims of sexual violence. This includes, but is not limited to, the extent to which jurors hold falsehoods, stereotypes, and gendered beliefs about male victims of sexual violence. We specifically consider this in the context of trials in England and Wales given the low conviction rates for rape and serious sexual offences in this jurisdiction (e.g., HM Government, 2022; Holh, 2022) and the fact that research on mock jurors’ perceptions of male victims is emergent in this context, meaning work with outcomes which specifically apply in England and Wales is needed to establish how jurors might make decisions in these cases. Overall, this understanding is important to, firstly, evaluate whether lay attitudes toward male victims of sexual violence translate to legal judgments and secondly, inform empirically grounded recommendations to address potential gender disparities in these judgments.

## Juror Perceptions of Victims of Sexual Offenses

Jurors and juries make a range of complex judgments when determining guilt, including the credibility, responsibility, and blame assigned to both defendants and complainants. Importantly, these judgments may rely on factors that are tangential to the case facts presented, especially in cases of sexual violence (Leverick, 2020). This can include preconceived and prejudicial ideas about how a typical rape victim behaves, which are known as rape myths.

More specifically, rape myths are “prejudicial, stereotyped, or false beliefs about rape, rape victims, and rapists” (M. R. Burt, 1980, p. 217) that have the effect of “minimising rape as a serious concern, blaming victims, and defending perpetrators” (Hogge & Wang, 2022, p. 422). One systematic review examining juries’ attitudes toward female rape victims found that rape myth acceptance (RMA) was significantly related to jurors’ attribution of a not-guilty verdict—regardless of the quality of the research study (e.g., validity and reliability of measures) and the sample used (e.g., student vs. nonstudent) (Dinos et al., 2015). Another review, drawing upon quantitative and qualitative research examining the effect of rape myths on mock jurors’ evaluation of evidence and decision-making in rape cases, found that these prejudicial and false beliefs had a significant impact on mock jurors’ decision-making (Leverick, 2020). More specifically, RMA predicted mock jurors’ attitudes about the responsibility and blame a female victim was ascribed for her assault—mock jurors with higher RMA scores were less punitive toward male defendants. Also, the qualitative results suggested that mock jurors often express attitudes about how “real” rape victims act and what a “real” rape looks like, during jury deliberations (Leverick, 2020).

Research suggests that jurors are also sensitive to extraneous factors that can influence their attitudes and beliefs in sexual violence cases—and may rely on these factors when determining their verdict (Lundrigan et al., 2019). This can include a victim’s physical characteristics and demeanor; for example, research suggests that a victim’s emotionality (i.e., showing distress) (Nitschke et al., 2019; Pals et al., 2024), attractiveness (Vrij & Firmin, 2001), and/or intoxication (Lynch et al., 2013; Martin & Monds, 2023), among other characteristics,^2^ can be influential in determining jurors’ attitudes toward witnesses as well as their verdict selections.

Research also shows that victim gender can play a significant role in making these determinations (see, e.g., Rye et al., 2006). Perceptions of sexual violence are gendered, and victims often encounter specific gender- and sex-role myths and stereotypes from community members (Carlisle & Schmitz, 2023; Weare, 2021; Weiss, 2010). For instance, in discussions of the “ideal victim,” one study suggested that “notions of victimhood show significant overlap with notions of (stereotypical) femininity” when individuals evaluate the likelihood that someone has been victimized (Bosma et al., 2018, p. 959). Stereotypes that relate to male victims of sexual violence also focus on the victim’s sexuality. Research suggests that mock jurors blame gay male victims more than straight and lesbian female victims (Davies et al., 2009; Wakelin & Long, 2003), and this effect is most pronounced among male mock jurors (Davies et al., 2009). Where a male victim’s sexual orientation suggests attraction to the perpetrator, they receive greater blame for their assault (Davies et al., 2006; Smith et al., 1988) as they are believed to receive more pleasure and less trauma from the assault (Mitchell et al., 1999).

However, despite findings that suggest men struggle to obtain legitimacy as victims of sexual violence (Widanaralalage et al., 2023), research is emergent in considering how mock jurors perceive male victims of sexual violence, where the sexual violence literature focuses almost exclusively on female victims’ experiences.^3^

## Public Perceptions of Male Victims of Sexual Offenses

On average, 9,000 men reported being victims of rape, or attempted rape, between 2009 and 2012 (Ministry of Justice, Home Office, & Office for National Statistics, 2013a), and across the same period, 1,276 instances of male rape (14%) were reported to the police (ONS, 2023). A total of 435 cases were subsequently prosecuted (ONS, 2018); 144 cases went to trial at Crown Court, and 90 defendants were found guilty—equating to a 62.5% conviction rate (Ministry of Justice, Home Office, & Office for National Statistics, 2013b)—yet a 1% conviction rate when considering the estimated rate of male rape during this period. This closely represents findings from empirical research. For example, one study found 12.5% of male rape victims surveyed reported their assault to the police, with only one defendant subsequently being convicted (Walker et al., 2005).

Men’s gendered experiences of sexual violence likely have implications for jury decision-making. Research suggests that male victims, including children (Drugge, 1992), are perceived as more responsible for their sexual assault than female victims (Gerber et al., 2004; but also see, D. L. Burt & DeMello, 2003) as they are expected to be able to physically resist their assailant (Davies et al., 2008). Lay decision-makers also perceive sexual assaults against men as less serious than those against women (Davies et al., 2001) and are less likely to label these assaults as “rape” or “sexual assault” (Gerber et al., 2004; Hannon et al., 2000). Male victims themselves are reluctant to label their sexual assault as a crime—with over one-third of men believing their assault was wrong, but not a crime (ONS, 2021). Research also suggests that people are more reluctant to recommend support to male victims following their assault, compared to female victims (Judson et al., 2013). This is in line with lay perceptions that sexual abuse is more severe and traumatic for female victims, compared to male victims (Bornstein et al., 2007). However, this is contrary to the actual harm that male victims experience from sexual violence. For example, almost 50% of men report developing mental or emotional problems, around 40% of men report physical injuries, and 1 in 10 men report having tried to commit suicide, following being raped or assaulted by penetration (ONS, 2021).

Research finds that participants are less likely to believe that men, particularly those assaulted by women, are victims of sexual violence, compared to women (Oswald & Russell, 2006). Individuals perceive sexual assaults involving male victims as less likely to have occurred, compared to those involving female victims, for both adult (Smith et al., 1988) and child victims of sexual assault (Drugge, 1992). This is important because beliefs about the plausibility of an event can contribute to beliefs about the veracity of a victim’s testimony. However, there are inconsistencies in the effects of victim gender on lay perceptions of victims, with some research suggesting that jurors perceive female victims more negatively than male victims (Anderson, 1999; Bornstein & Muller, 2001; Schneider et al., 1994) and other research finding that victim gender does not affect perceptions of a victim’s believability (see, e.g., Cromer & Freyd, 2007).

The extent to which beliefs and attitudes toward male victims influence jurors’ verdict decisions is important, particularly in the context of sexual offenses where, in the absence of corroborating evidence (e.g., physical evidence or eyewitness testimony), trial outcomes depend heavily on jurors’ perceptions of the complainant and defendant, such as how credible, honest, or believable they perceive them to be (e.g., Mcintosh & Davis, 2022; Willmott et al., 2018). It is therefore important to explore how mock jurors’ perceptions of a complainant and defendant translate to verdict decisions, as well as understand the extent to which jurors rely on assumptions and falsehoods in their determinations of guilt. This is not only important in ensuring that jurors fulfill their role effectively (e.g., in upholding normative principles of the criminal legal system such as fairness and equality), but also because negative reactions from jurors have the potential to act as a form of secondary victimization (e.g., J. E. Williams, 1984).

However, in England and Wales, legislative restrictions prevent enquiry into any aspect of jury deliberations. Mock jury studies are therefore an effective means of gaining insight into how jurors deliberate and reach a verdict. While mock jury studies are a well-established means of understanding how jurors make decisions, we acknowledge that this methodology is limited in terms of its ecological validity and subsequent ability to reflect real juror judgments. Despite this, mock jury studies that experimentally manipulate case-related variables (e.g., victim gender) to predict legal judgments (e.g., verdict) allow a high degree of control over decision-making conditions to allow for a nuanced understanding of the relationship between these factors.

## Summary

In this paper, we systematically review the literature on juror decision-making in sexual offense cases with a male victim. We seek to understand how mock jurors’ attitudes toward male victims translate to verdict outcomes in criminal trials and understand the extent to which these decisions reflect lay perceptions of male victims more widely, in terms of mock jurors’ use of male rape myths. To understand how victim gender affects mock jury decisions, we aim to discern differences in mock jurors’ perceptions of, and verdicts in cases with, male victims of sexual offenses, compared to female victims. We also seek to explore how mock jurors use a defendant’s gender to inform their decisions in sexual offense cases with male victims, particularly when the defendant is female.

## Method

This systematic review was conducted consistent with guidelines from the Preferred Reporting Items for Systematic Reviews and Meta-Analyses (PRISMA-2020; Page, McKenzie, et al., 2021, Page, Moher, et al., 2021). It was also preregistered on the Open Science Framework (OSF), see https://osf.io/tz98h, using the Generalized Systematic Review Registration Form (van den Akker et al., 2023).

### Eligibility Criteria

The publications were included if they were peer-reviewed, experimental studies published between 1980 and 2024.^4^ Due to resource restrictions, articles were required to be published in English. The publications were included if they examined mock *juror* or *jury* assessments of guilt (defined as a binary verdict, or continuous guilt rating) in a case involving an adult male victim of a sexual offense (i.e., rape, sexual assault, or causing sexual activity without consent).^5^

The publications were excluded from this review if they (a) were not in a criminal trial context (e.g., in a civil trial context, see McCracken & Stevenson, 2017), (b) were not set in a mock trial or mock jury context (i.e., assessed attitudes of lay decision-makers more generally), (c) did not include an assessment of guilt (e.g., binary verdict or continuous assessment of the likelihood that the defendant committed the offense, see e.g., Davies & McCartney, 2003), or (d) did not include legal decision-making which is relevant to the criminal trial context in England and Wales (i.e., death-penalty decision-making, see Girgenti, 2015; M. R. Williams et al., 2007). Exclusion criteria a-d were chosen to ensure high ecological validity and generalizability to a criminal trial context in England and Wales.

The publications were also excluded from this review if they (e) were not peer-reviewed and not experimental (e.g., a dissertation or thesis, see Schueller, 2022), (f) did not include a male victim (however, publications were still included if victim gender was manipulated to compare male and female victims), (g) did not include a sexual offense (e.g., rape, sexual assault, or causing sexual activity without consent) (i.e., sexually motivated, violent offenses, see Rye et al., 2006), (h) did not include a cisgender victim (i.e., transgender or nonbinary victims alone) to avoid confounding effects (e.g., Michalski et al., 2022),^6^ or (i) did not include an adult victim (i.e., children, see Quas et al., 2002). Exclusion criteria e-i ensure a clear focus on the research question and avoid effects that can confound how mock jurors perceive male victims.

### Information Sources and Search Strategy

The following 3 interfaces, which contained 11 databases in total, were searched: EBSCOhost, which included Academic Search Ultimate, APA PsycArticles, APA PsycInfo, British Education Index, Child Development & Adolescent Studies, MEDLINE Complete, ERIC, Educational Administration Abstracts, and SocINDEX. PubMed and Scopus were also searched. The searches were conducted in June 2024. The following search strings were used across all interfaces:

1. (((Victim/ Complainant) + (Gender/Sex)) + (Sexual assault/ rape/ sexual abuse))
e.g., “victim gender” AND “sexual assault”^7^
2. (((Male) + (Victim/ Complainant) + (Sexual assault/ rape/ sexual abuse))
 e.g., “male complainant” AND “rape”

To complement these searches, both ascendancy (searching included sources for sources cited) and descendancy approaches (searching for sources that cite included sources) were employed to search for additional, relevant literature. To locate gray literature, a final exploratory Google Scholar and Web of Science search was conducted across June and July 2024.^8^

### Selection Process and Data Collection

Sources were screened in three stages: (a) title-only, (b) abstracts and keywords, and (c) full-texts. One reviewer (MM) independently screened records to determine they met the preregistered inclusion criteria for the review; those that did not were removed. No studies were excluded other than for not meeting the pre-registered exclusion criteria. In the first round of screening, blinding was used, whereby only the title of the source was available, to avoid bias. References were collated using Zotero v.6.0.36. One reviewer (MM) also independently extracted relevant data from records. Data were collated using Microsoft Excel.

### Data Items and Effect Measures

The main independent variable of interest is victim gender. Other independent variables of interest include defendant gender, juror gender, victim sexuality, and the type of sexual offense described in the case facts. The main dependent variable of interest is mock jurors’ binary verdict, measured by a significant difference (*p* < .05) in guilty and not guilty verdicts between cases which involve a male victim and those which involve a female victim, or between cases which manipulate a male victim’s demographic characteristics (e.g., Black vs. White male victims). Other dependent variables of interest include continuous assessments of guilt (e.g., certainty or confidence in the defendant’s guilt), other legally relevant outcomes (e.g., punishment assigned), and mock jurors’ assessments of the victim and defendant.

### Quality Assurance

The sources were appraised in terms of quality using The Quality of Survey Studies in Psychology (Q-SSP; Protogerou & Hagger, 2020). This is a generic measure that assesses the quality of sources across four domains: introduction, participants, data, and ethics. This measure of quality assessment is also supplemented by eight domain-specific criteria related to jury decision-making, devised by the authors: (a) Sample: Representative sampling pool, (b) Sample: Jury eligible, (c) Sample: Power analysis, (d) Judgments of guilt, (e) Presentation of materials, (f) Manipulation checks, (g) Outcome measure, and (h) Alternative outcome measure reliability. Full details of these eight items, including how they were coded, can be found in the preregistration.

Three reviewers (MM, SW, and DW) extracted quality assessment data independently. All reviewers reconvened to evaluate agreement on the quality assessment items (measured using Cohen’s Kappa), with any discrepancies resolved via discussion. If an agreement could not be reached, a majority decision (two of three reviewers agreed) was used to make the final determination. No sources were removed due to quality. Two independent quality assessment scores were calculated. Final scores were agreed upon by all three reviewers.

### Data Synthesis

Given the heterogeneity between the sources and outcome measures, no data transformations were performed, and a meta-analysis was not undertaken. The results of this systematic review are reported descriptively. The synthesis of data was performed by one reviewer (MM), with any uncertainty discussed with two other reviewers (SW and DW).

## Results

### Study Selection

**Figure 1.**
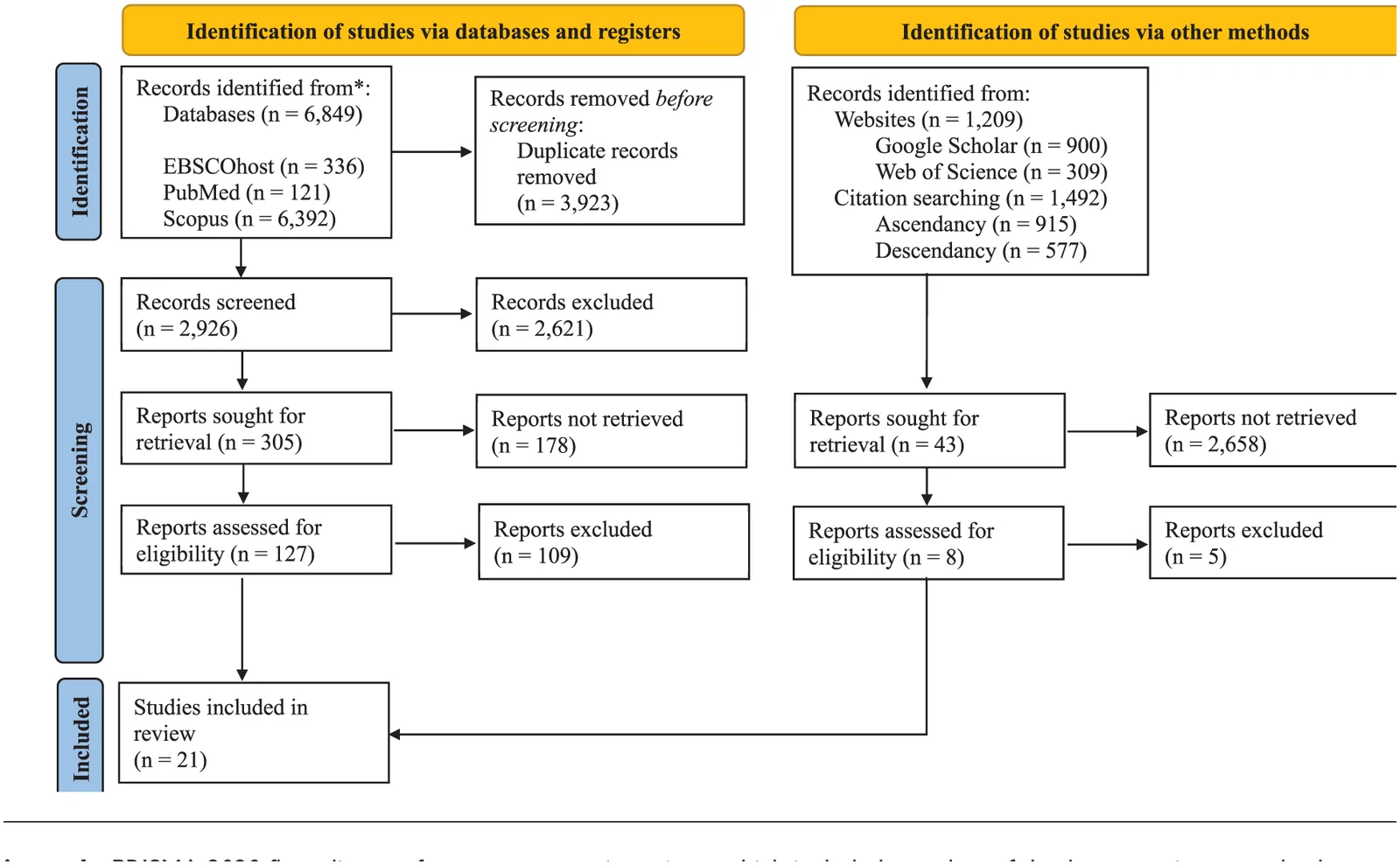
PRISMA 2020 flow diagram for new systematic reviews which included searches of databases, registers, and other sources. *Source*. Page, McKenzie, et al. (2021). *Note*. PRISMA = Preferred Reporting Items for Systematic Reviews and Meta-Analyses.

The initial interface searches returned 6,849 results. A total of 3,923 sources were removed as they were duplicates, and accordingly, 2,926 records were included for extraction. Following the first round of extraction using titles-only, 2,621 records were excluded, and after the second round of extraction using abstracts and keywords, 178 records were excluded. Finally, in the third round of extraction, one author (MM) read the remaining 127 records in their entirety, resulting in the exclusion of 109 sources. This left 18 records to be included for synthesis—one source contained four studies, two of which fit the preregistered inclusion criteria (Klement et al., 2019, Study 3, Study 4). Ascendancy and descendancy searches revealed one further source (Seaman et al., 2001), and gray literature searches revealed a further two sources (Starosta et al., 2024; Starosta & Schuller, 2020). Overall, a total of 21 sources were included for synthesis, see Figure 1 for an overview.

### Study Characteristics and Diversity

The 21 reviewed studies were heterogeneous in their measured and outcome variables, for a summary see Tables 1–3. Geographically, 5% (*N* = 1) were conducted in the United Kingdom, 24% (*N* = 5) in Canada, and 71% (*N* = 15) in the United States.

**Table 1.** Final Extracted Publications: Mock-jurors Become Less Punitive.

Study	Defendant Gender	Complainant Gender	Assessment of Guilt	Other Dependent Variables	Quality Assessment Score (Jury Specific Score/ Q-SSP Score)
Klement et al., 2019, Study 3	Female	Male	Continuous	MMRMS	1/ 65%
Klement et al., 2019, Study 4	Female	Male	Continuous	MMRMS	1/ 65%
Moore & Miller-Perrin, 2022	Manipulated	Male and female	Continuous	Negative emotional impact (e.g., shame, guilt, embarrassment, and trauma)	2/ 70%
Russell et al., 2011	Manipulated	Male and female	Binary and continuous	Legal elements associated with sexual assault	1/ 45%
Sommer et al., 2016	Manipulated	Male and female	Binary	Mini-K and Rape Myth Acceptance Scale	4/ 60%
Starosta et al., 2024	Manipulated	Male and female	Binary	Prototypicality of the case, IRMAS, and Male Rape Myth Acceptance Scale	5/ 80%
Seaman et al., 2001	Male	Male and female	Continuous	Believability	0/ 35%

*Note*. MMRMS = Melanson’s Male Rape Myth Scale; IRMAS = Illinois Rape Myth Acceptance Scale; Q-SSP = Quality of Survey Studies in Psychology.

**Table 2.** Final Extracted Publications: Mock-jurors Do Not Become More or Less Punitive.

Study	Defendant Gender	Complainant Gender	Assessment of Guilt	Other Dependent Variables	Quality Assessment Score (Jury Specific Score/ Q-SSP Score)
Carter et al., 2023	Male	Male and female	Binary and continuous	Crime severity	4/ 70%
Hafer & Jacquin, 2022	Manipulated	Male and female	Binary and continuous	Feelings toward the U.S. military, military women, and military men, Ambivalent Sexism Inventory, AMMSA, and views on whether the act was consensual sex or rape	5/ 85%
Levi et al., 2024	Male	Male and female	Binary	Perceived sexual desire, sympathy toward, and perceived responsibility of the victim and defendant.	5/ 80%
Mcintosh & Davis, 2022	Male	Male and female	Binary	AMMSA Scale, Homosexuality Attitude Scale, and Just World Scale	4/ 80%
Mitchell et al., 2009	Male	Male and female	Continuous	Marlowe–Crowne Social Desirability Scale	0/ 35%
Pals et al., 2024	Male	Male and female	Binary and continuous	Not Applicable	6/ 75%
Pica et al., 2020a	Male	Male and female	Binary and continuous	IRMAS	4/ 50%
Pica et al., 2021	Manipulated	Male and female	Binary and continuous	IRMAS	3/ 55%
Smith et al., 1988	Manipulated	Male and female	Continuous	Likelihood of act	0/ 25%
Starosta & Schuller, 2020	Manipulated	Male and female	Binary	Rape Myth Acceptance Scale	4/ 55%

*Note*. IRMAS = Illinois Rape Myth Acceptance Scale; Q-SSP = Quality of Survey Studies in Psychology; AMMSA = Acceptance of Modern Myths about Sexual Aggression.

**Table 3.** Final Extracted Publications: Mock-jurors Become More Punitive.

Study	Defendant Gender	Complainant Gender	Assessment of Guilt	Other Dependent Variables	Quality Assessment Score (Jury Specific Score/ Q-SSP Score)
Ellingwood et al., 2023	Not specified	Male and female	Binary and continuous	Lesbian, Gay, and Bisexual Knowledge and Attitudes Scale for Heterosexuals and International Rape Myth Acceptance Scale	3/ 45%
Hill, 2000	Male	Male and female	Binary and continuous	Not Applicable	1/ 30%
Pica et al., 2020b, Study 2	Male	Male and female	Binary and continuous	Perceptions of eyewitness	3/ 45%
Powers et al., 2023	Male	Male	Continuous	Homophobia, racism, and racism (sexual assault)	3/ 60%

*Note*. Q-SSP = Quality of Survey Studies in Psychology.

### Quality Assessment

Three reviewers (MM, SW, and DW) assessed the quality of all 21 sources using two separate measures. The results of these assessments are provided in Tables 1–3.

### Results of Individual Studies

#### Independent Variables

Forty-eight percent (*N* = 10) of sources used only male defendants in their vignette, 10% (*N* = 2) used only female defendants in their vignette, and 38% (*N* = 8) of studies manipulated the gender of the defendant. One study did not mention the gender of the defendant (Ellingwood et al., 2023). 86% (*N* = 18) of studies manipulated the gender of the victim, and the remaining 14% (*N* = 3) of studies used only male victims. Forty-three percent (*N* = 9) of sources controlled for the effects of participant gender; the remaining sources did not. The offense specified was heterogeneous between sources. Fifty-two percent of sources (*N* = 11) specified the offense tried as a sexual assault, and 38% (*N* = 8) specified it as rape. One study specified the offense as sodomy (Levi et al., 2024), and one study manipulated the offense specified to be either vaginal sex or an oral assault (Starosta et al., 2024), see Tables 1–3 for a summary.

#### Dependent Variables

Twenty-four percent of sources (*N* = 5) measured guilt using a binary verdict alone (i.e., guilty or not guilty), and 33% of sources (*N* = 7) measured guilt using a continuous measure. The remaining 43% of sources (*N* = 9) used both types of measure, see Tables 1–3 for a summary. Forty-eight percent of sources (*N* = 10) also asked mock jurors to suggest a sentence or assign a form of punishment if a defendant was found guilty. No sources included a form of deliberation between mock jurors, although 10% of sources (*N* = 2) asked participants to provide a reason for their verdict. Thirty-eight percent of sources (*N* = 8) measured mock jurors’ belief in rape myths, and 14% (*N* = 3) measured mock jurors’ belief in male rape myths more specifically.

### Results of Syntheses

#### Verdicts in Cases of Rape and Sexual Assault with a Male Victim

##### Mock Jurors Become Less Punitive

Thirty-three percent of sources (*N* = 7) found that fewer guilty verdicts were assigned to a defendant when the complainant was male, compared to when the complainant was female. Five sources directly manipulated victim gender (Moore & Miller-Perrin, 2022; Russell et al., 2011; Seaman et al., 2001; Sommer et al., 2016; Starosta et al., 2024). Across all five studies, mock jurors were less punitive toward defendants in cases of sexual violence with a male victim, compared to those with a female victim.

For instance, Moore and Miller-Perrin (2022) manipulated defendant and victim gender to examine mock jurors’ decision-making in a sexual assault case. They found that, regardless of mock jurors’ own gender, participants perceived a male defendant as more guilty when they were alleged to have sexually assaulted a female victim, compared to when a female defendant was alleged to have sexually assaulted a male victim (Moore & Miller-Perrin, 2022). One study found that the odds of mock jurors voting guilty increased by 132.7% for a male defendant in a case with a female victim compared with a female defendant in a case with a male victim (Sommer et al., 2016).

Two studies examined the effect of strategies to address male rape myths on mock jurors’ verdict decisions in rape cases with a male victim and female defendant (Klement et al., 2019, Study 3, Study 4). Across both studies, the authors found that higher male RMA among mock jurors was associated with lower perceived defendant guilt (the correlation coefficients for these variables in Studies 3 and 4 were −.52 and −.62, respectively, both of which were significant at *p* < .001)

##### Mock Jurors Do Not Become More or Less Punitive

Forty-eight percent of sources (*N* = 10) found no difference in guilty and not guilty verdicts assigned to a defendant when the victim was male, compared to the comparison group. While the majority of studies found no effect of victim gender across any guilt measures or mock juror judgments or ratings (Carter et al., 2023; Hafer & Jacquin, 2022; Mcintosh & Davis, 2022; Mitchell et al., 2009; Pica et al., 2020b), five studies found that, despite no significant effect of victim gender on verdict, mock jurors perceived male victims more negatively, and defendants in cases with a male victim more favorably, compared to cases with female victims (Levi et al., 2024; Pals et al., 2024; Pica et al., 2021; Smith et al., 1988; Starosta & Schuller, 2020).

Mock jurors viewed defendants as more credible (Pals et al., 2024) and female defendants in particular as more in control (Pica et al., 2021) when the victim was male, compared to when the victim was female. Mock jurors also perceived male victims less favorably (Pica et al., 2021), viewed them as less credible (Levi et al., 2024), and blamed them more than female victims (Pals et al., 2024). When the defendant was female, she was perceived as more in control (Pica et al., 2021) and the male victim was seen as more encouraging, deriving more pleasure, and experiencing less stress (Smith et al., 1988) compared to when the victim was female. Mock jurors also perceived the likelihood of the sexual assault as higher when the victim was female compared to male (Smith et al., 1988).

##### Mock Jurors Become More Punitive

Nineteen percent of sources (*N* = 4) found that more guilty verdicts were assigned to a defendant when the victim was male, compared to a comparison group. Of these sources, only one found an effect in terms of victim gender. Pica et al. (2020a) examined the effects of several factors, including type of assault, victim-defendant familiarity, the extent of delayed reporting, and victim gender, on mock juror judgments and beliefs. The authors found that mock jurors attributed higher guilt ratings to the defendant when the victim was male, compared to when the victim was female. However, there were no effects of victim gender on verdict or on perceptions of either the victim or defendant (Pica et al., 2020a).

#### Victim Characteristics

The remaining studies examined the effect of specific victim characteristics (e.g., race, sexuality, and gender identity) on mock juror judgments. In particular, mock jurors were most sure of a defendant’s guilt in intraracial cases of sexual violence but were less certain in interracial cases (Powers et al., 2023). Mock jurors were also less punitive toward defendants in cases with transgender male victims, compared to both male and female cisgender victims (Ellingwood et al., 2023), and were also less punitive toward defendants in cases with homosexual victims than defendants in cases with heterosexual victims—in particular where the victim was a homosexual man (Hill, 2000). Overall, these studies suggest that male victims can possess specific characteristics that trigger lenient mock juror decisions for defendants in sexual offense cases, such as their race, sexuality, and gender identity.

##### Race

One study examined a sexual assault scenario involving two male college athletes, whereby the race of the defendant and victim was manipulated to examine effects on mock jurors’ perceptions of guilt and attitudes (Powers et al., 2023). Mock jurors believed that, compared to White victims, Black victims in cases with a White defendant should have been able to physically resist the assault more, and that failing to so made them partially responsible for their assault. The authors also measured mock jurors’ racist attitudes, both broadly and specifically related to sexual assault (e.g., measuring the extent to which racial minorities are criminogenic), and found that racism was associated with a belief that harm to male victims from sexual assault is exaggerated, and that men should be able to physically resist a sexual assault. Both measures were associated with the belief that male victims of sexual assault are typically gay (Powers et al., 2023).

##### Sexuality

Three studies manipulated victim sexuality alongside victim gender (Ellingwood et al., 2023; Hill, 2000; Levi et al., 2024). Mock jurors were more sympathetic toward heterosexual male victims (Levi et al., 2024) and more likely to find the defendant guilty when the victim was heterosexual, compared to when they were homosexual (Hill, 2000). In particular, mock jurors were more likely to find homosexual men accused of sexually assaulting heterosexual men guilty than either heterosexual men accused of assaulting heterosexual women or homosexual men accused of assaulting other homosexual men (Hill, 2000).

One study found no effect of victim sexuality on verdicts, perceptions of guilt, or perceptions of either the defendant or victim (Ellingwood et al., 2023). However, the authors also assessed mock jurors’ endorsement of knowledge and attitudes toward the LGB community using the Lesbian, Gay, and Bisexual Knowledge and Attitudes Scale for Heterosexuals (LGB-KASH). Mock jurors who scored highly on the LGB-KASH hate subscale (e.g., “It is important for me to avoid LGB individuals”) had more positive perceptions of the defendant and attributed lower guilt ratings to the defendant. On the other hand, mock jurors who scored highly on the civil rights subscale (e.g., “Hospitals should acknowledge same-sex partners equally to any other next of kin”) had less favorable views of the defendant and assigned higher guilt ratings to the defendant. Only the knowledge subscale (e.g., “I am knowledgeable about the history and mission of the PFLAG organization”) was associated with a dichotomous verdict, albeit counterintuitively—mock jurors with higher scores on this subscale were less likely to determine the defendant to be guilty (Ellingwood et al., 2023).

Two sources also measured mock jurors’ homophobic beliefs (Mcintosh & Davis, 2022; Powers et al., 2023). Male mock jurors typically displayed more homophobic attitudes than female mock jurors (Mcintosh & Davis, 2022). Homophobic attitudes were associated with prejudicial attitudes, correlating strongly with RMA (Mcintosh & Davis, 2022) and attitudes toward victims, with higher homophobic attitudes predicting higher perceptions of victim culpability (Powers et al., 2023), as well as predicting verdict decisions (Mcintosh & Davis, 2022). In particular, the effect of homophobia on verdicts varied based on victim gender; counterintuitively, homophobia did not predict verdicts in cases with a male victim. In cases with a female victim, homophobia was associated with fewer guilty verdicts (Mcintosh & Davis, 2022).

##### Gender Identity

While the preregistered inclusion criteria for this review specified a cisgender male victim, two studies manipulated the gender identity of the victim to compare mock jurors’ attitudes toward cisgender and transgender victims and were therefore eligible for inclusion (Carter et al., 2023; Ellingwood et al., 2023). Mock jurors were generally less punitive toward defendants in cases with transgender male victims, compared to both cisgender victims (Ellingwood et al., 2023) and transgender female victims (Carter et al., 2023). However, the effects of victim gender identity on verdicts were inconclusive. While one study found that mock jurors were more likely to find a defendant guilty when the victim was a cisgender man or a cisgender woman compared to a transgender man (Ellingwood et al., 2023), another found no effect of gender identity on verdicts (Carter et al., 2023).

#### Defendant Gender

While the majority of studies in this review investigated the effect of a male defendant, some studies also manipulated the gender of the defendant, in addition to the gender of the victim. Generally, the studies found that mock jurors perceived male defendants, accused of assaulting a female victim, as more guilty than female defendants accused of assaulting a male victim (Moore & Miller-Perrin, 2022; Russell et al., 2011; Sommer et al., 2016), with male defendants also receiving longer sentences than female defendants (Smith et al., 1988; Sommer et al., 2016).

Mock jurors’ attitudes toward victims also varied based on defendant gender. When the defendant was female, and the victim male, mock jurors were more likely to assume that the victim consented, was less likely to be injured (Russell et al., 2011), encouraged the act, experienced less stress, derived more pleasure (Smith et al., 1988), and bore greater responsibility (Sommer et al., 2016; Starosta et al., 2024; Starosta & Schuller, 2020) compared to when the defendant was male, and the victim female.

#### Juror Gender

Several sources also explored the effect of mock juror gender on outcomes. Generally, male mock jurors displayed fewer pro-victim attitudes than female mock jurors (e.g., Pals et al., 2024). The sources suggested that men blamed the victim more (Carter et al., 2023; Mitchell et al., 2009), believed that the victim was more responsible (Levi et al., 2024) and less credible (particularly in terms of male victims) (Starosta & Schuller, 2020), and thought that the victim encouraged the act more (Smith et al., 1988), compared to women. Despite these differences, several sources found that gender differences in mock jurors’ attitudes toward victims and defendants did not translate into verdicts (however, see Mcintosh & Davis, 2022), with no effect of participant gender on mock jurors’ binary verdicts (Carter et al., 2023; Moore & Miller-Perrin, 2022; Pals et al., 2024; Smith et al., 1988).

#### Juror Rape Myth Acceptance

Several sources explored the effect of female (Ellingwood et al., 2023; Hafer & Jacquin, 2022; Pica et al., 2020a, 2020b, 2021; Starosta et al., 2024; Starosta & Schuller, 2020) male (Klement et al., 2019; Starosta et al., 2024), and gender-neutral rape myths (Sommer et al., 2016) on mock jurors’ attitudes in cases with a male victim. Individual differences were reported in RMA, with male mock jurors reporting higher RMA scores compared to female mock jurors (Starosta & Schuller, 2020). In terms of effects on decision-making, RMA, measured using the Illinois Rape Myth Acceptance Scale (IRMA), predicted verdict decisions (mock jurors with lower RMA scores were more likely to find the defendant guilty) as well as guilt ratings (mock jurors with lower RMA scores were more likely to attribute higher guilt ratings to the defendant) (Pica et al., 2020a, 2021). Higher scores on the Acceptance of Modern Myths about Sexual Aggression scale also predicted lower ratings of defendant guilt (Hafer & Jacquin, 2022). However, neither source discriminated between assessments of guilt in cases involving male versus female victims. Sources that used different rape myth scales (the International Rape Myth Acceptance Scale and a gender-neutral Rape Myth Acceptance Scale, respectively) found no effect of RMA on verdict decisions (Ellingwood et al., 2023; Sommer et al., 2016).

One source investigated the effect of both measured male and female rape myth acceptance on mock jurors’ attitudes (Starosta et al., 2024). The researchers found divergent effects in terms of each measure; while mock jurors with high IRMA scores attributed blame to a female victim to a greater extent than a male victim, mock jurors with high MRMA scores attributed blame to a male victim to a greater extent than a female victim. While generally MRMA scores were low, any acceptance of male rape myths predicted negative perceptions of the male victim (Starosta et al., 2024).

Two studies explored the potential immutability of mock jurors’ male rape myths. Klement et al. (2019, Studies 3 and 4) presented mock jurors with either confirming or debunking information about male rape myths to evaluate the impact on mock jurors’ judgments of a male victim and female defendant, as well as understand whether intervention success varied based on mock jurors’ male RMA. The authors found that higher male RMA was associated with greater perceived culpability and pleasure of the victim, lower credibility and trauma of the victim, and lower perceived defendant guilt. The authors also found that mock jurors with high male RMA scores were particularly unaffected by information that aimed to debunk male rape myths, compared to those with lower male RMA scores (Klement et al., 2019), see Tables 4 and 5 for a summary of critical findings and implications for practice, policy, and research, respectively.

**Table 4:** Critical Findings.

Factor	Key Findings
Complainant gender	The majority of sources suggest that complainant gender does not affect on mock jurors’ assessments of guilt.
Complainant characteristics	Male complainants possess specific vulnerabilities which can affect punitiveness among mock jurors (defendants in cases with Black, gay, transgender male complainants are treated less punitively than defendants in cases with white, heterosexual, or cisgender complainants).
Defendant gender	Mock jurors were generally more punitive toward male defendants, compared to female defendants. They also hold fewer pro-victim attitudes when the defendant is female, and the complainant is male—although these do not translate to assessments of guilt.
Juror gender	Male mock jurors generally displayed fewer pro-victim beliefs than female mock jurors, although these often did not translate to verdicts.
RMA	RMA was associated with fewer pro-victim attitudes—although did not always affect guilt ratings. It is important that research discriminates between male and female rape myths, particularly as RMA more generally can be difficult to ameliorate.

*Note*. RMA = Rape Myth Acceptance.

**Table 5.** Practice, Policy, and Research Implications.

Implication Type	Recommendations
Practice and policy	Provide evidence-based training to jurors that addresses male rape myth acceptance, particularly in cases with a male complainant. This is in light of research that suggests that judicial instructions may be ineffective at ameliorating these specific myths.
Research	Conduct ecologically valid jury research that specifically examines the experiences of male victims. More specifically, research should also examine how gender interacts with other characteristics such as ethnicity, sexuality, and gender identity to understand how these factors present additional vulnerabilities to male victims in the criminal legal system.

## Discussion

This systematic review is the first to examine mock jurors’ determinations of guilt in sexual offense cases involving male victims—specifically by investigating the effect of victim gender on verdict decisions. Understanding mock jurors’ perceptions of male victims in cases of sexual violence is important because men have specific gendered experiences, and research is emerging in understanding how mock jurors translate lay perceptions to verdicts. For example, whether mock jurors use falsehoods and stereotypes related to male victims’ experiences of sexual violence, in the same way as research suggests they do in cases of female sexual victimization (e.g., Leverick, 2020).

The results of this systematic review suggest that the relationship between victim gender and mock jurors’ determinations of guilt and attitudes is nuanced. For instance, nearly half of the sources found that victim gender did not affect mock jurors’ guilt ratings. However, several of these sources did find that mock jurors’ perceptions of male victims were more negative than the comparison group, in line with expectations surrounding RMA (e.g., Klement et al., 2019, Study 3, Study 4). Almost a quarter of sources found that victim gender reduced punitiveness toward defendants, whereby mock jurors were less likely to assign guilty verdicts or rate the defendant as guilty when the victim was male compared to female. Levi et al. (2024) suggested that the absence of a difference in verdicts between straight and gay male victims may have been due to differences in attitudes that nonetheless produced the same direction in verdicts (i.e., straight male victims provoke male rape myths, such as being perceived as more masculine and able to fight off an attacker, resulting in more not-guilty verdicts, and gay male victims provoke “heterosexist biases” which suggest they desired and consented to the sexual act, resulting in more not-guilty verdicts) (Levi et al., 2024, p. 9). This links to research which suggests that decision-makers’ perceptions of the victim’s experience of the assault (e.g., whether they experience distress) impact whether the act is viewed as criminal and therefore worthy of attributing guilt (Catton & Dorahy, 2022).

Yet, some sources found the opposite effect. Almost one-fifth of the sources found that the defendant was in fact perceived as more guilty when the victim was male—although the only study to directly compare male and female victims found this effect in respect to continuous verdict assessments, but not dichotomous verdicts. This suggests that again, mock jurors did not perceive their attitudes as influential enough to convict the defendant. This can be interpreted in the context of RMA whereby decision-makers rely heavily on any opportunity to exonerate a perpetrator, despite a significant belief in their guilt (e.g., Bohner et al., 2009). In particular, this review highlights that a male victim’s ethnicity, sexual orientation, and gender identity are influential in determining verdicts in cases of sexual violence; mock jurors held fewer pro-victim attitudes toward Black men, compared to White men, toward gay men, compared to heterosexual men, and toward transgender men, compared to cisgender men—and relatedly, defendants were treated less punitively in these cases. This finding is important, particularly where research suggests that 27% of Black and minority ethnic men who have sex with men in the United Kingdom report experiencing sexual abuse (i.e., any nonconsensual sexual contact) (Jaspal et al., 2017). Given that research also suggests that Black and Asian men experience specific barriers to disclosure and help-seeking that stem from cultural norms (Widanaralalage et al., 2024), this illustrates the importance of understanding jurors’ attitudes toward male victims of sexual violence through an intersectional lens (Crenshaw, 2015).

This review also suggests that jurors’ attitudes are important in determinations of guilt in cases where men are victims of sexual violence. In particular, mock jurors with higher RMA scores held fewer pro-victim attitudes and were less likely to assign a guilty verdict—interestingly, this effect depended on the type of RMA scale used. More specifically, only one source examined male RMA, finding that mock jurors with higher male RMA scores were more likely to hold fewer pro-victim attitudes, compared to those with lower male RMA scores. However, further research is needed that examines gender-specific rape myths in cases where the gender of the victim and/or defendant is varied. Understanding the impact of rape myths on jurors’ decisions in cases with male victims of sexual violence is important as research suggests that interventions to address these false and prejudicial beliefs can be successful in predictable circumstances (e.g., training on empathizing with victims, judicial directions) (Hudspith et al., 2023, 2024).

### Limitations

#### Limitations of Evidence

These results, and their implications, must be considered in light of several limitations of the sources reviewed. In particular, very few studies accurately represented real jury service; for example, none required jurors to deliberate before reaching a verdict. Deliberation serves an important function by providing reassurance and accountability to jurors’ individual verdict preferences, while also facilitating a shift toward a collective final verdict (Ellison & Munro, 2010). Therefore, researchers argue that one of the greatest threats to jury decision-making research is the reliance on nondeliberating mock juries, and associated deficits in ecological validity (Nuñez et al., 2011). Crucially, further research is needed which accurately reflects cases of sexual violence involving male victims, employs representative sampling techniques to select mock jurors, and allows for deliberation before reaching a final verdict (see, Willmott et al., 2021, for a summary of six methodological criteria that jury research should meet).

Ecological validity was also compromised in a number of studies where the vignettes were not tailored to the gendered experience of male sexual violence—for example, statistics suggest that men, compared to women, are more likely to be assaulted by a stranger (42.6% vs. 14.9%; Table 1), and the assault is less likely to happen in the man’s own home (21.7% vs. 37.9%; Table 8) (ONS, 2021). Research has noted the importance of understanding the balance of experimental control and realism—and where studies “prioritis[e] internal validity, ecological may [be] sacrificed because the vignettes [are] not tailored to suit the typical setting of each type of rape” (Sommer et al., 2016, p. 2860).

#### Limitations of Review Processes

Implications for practice, policy, and research should also be tentatively considered in light of the limitations of the processes used in this review. For example, the review only considered studies that reported a verdict decision, meaning that several studies on male sexual victimization and lay decision-making more broadly were excluded (e.g., Davies et al., 2001; Davies & McCartney, 2003). Due to resource constraints, sources were limited to English-language peer-reviewed publications (and therefore, academic theses were not included). Future reviews on this topic should consider broadening the search to include a more diverse range of sources.

## Conclusion

The present systematic review concludes that, generally, victim gender does not impact jury verdicts. However, while not exerting a tangible effect on verdicts, several sources suggest that mock jurors are less punitive toward defendants in sexual violence cases with male victims, compared to those with female victims—as well as reporting harsher attitudes and beliefs toward male victims. Research also suggests that specific characteristics can affect male victims’ access to fair outcomes in the criminal trial. In particular, ethnicity, gender identity, and sexual orientation are important determinants of juror attitudes, beliefs, and decisions in cases of sexual violence with a male victim—and mock jurors display fewer pro-victim attitudes and less punitive attitudes toward the defendant in these cases. The results from this review tentatively suggest that evidence-based training and educational strategies for jurors may be important in reducing stereotypes and misconceptions held about male victims of sexual violence, but crucially, further research is needed that more accurately reflects real jury decision-making, as well as how male victims experience sexual violence.

## Notes

1. The language used to describe individuals who have experienced, or are alleged to have experienced, sexual violence has been carefully considered. Using recommendations from a paper presented at the College of Policing Professional Committee (who consulted with a range of practitioners, charities, legal professionals, and academics to discuss the terminology used in sexual violence cases), we use the term “victim” to describe individuals who report instances of sexual violence perpetrated against them. However, when discussed in the context of the criminal legal system, we use the term “complainant.” We acknowledge that some preferred certain terms (e.g., survivor), while others advocate for any labels to be avoided (Beckley, 2018).

2. The range of factors identified to illustrate the extent to which jurors rely upon prejudicial information at the expense of probative evidence reflects the state of the literature, rather than the authors’ determination that these are the most important factors which influence jury decision-making in sexual violence cases.

3. This reflects the fact that women are disproportionately affected by sexual violence. Yet, it is also important to note that significant stigma exists toward male victims of sexual violence which prevents their victimization; for example, when the woman is a perpetrator, from being reported in official statistics (i.e., often men do not report their sexual victimization to anyone, and only certain types of sexual offending have data reported on them), see Weare (2020).

4. We selected the criterion that papers should be published from 1980 onward, so as to draw upon as much research as possible in this underresearched area. While we acknowledge that attitudes toward male victims have changed over the past 45 years in ways that may affect mock juror decision-making, research examining the prevalence of male rape myths among the general population demonstrates that acceptance of these myths remains pervasive in the United Kingdom (Weare & Willmott, In Preparation).

5. We acknowledge that different jurisdictions use different definitions for sexual offenses. This review relies on definitions provided within the Sexual Offences Act 2003, where rape is defined as the nonconsensual “penetration of the vagina, anus or mouth by a penis”, sexual assault is defined as “intentionally touching a person sexually without their consent” and sexual activity without consent refers primarily in this context to where “a complainant is forced to engage in sexual activity with the offender e.g. woman forces a man to penetrate her”.

6. Systematic research has recently been conducted on the effects of gender identity on jurors’ attitudes in a sexual assault case (see, Ribeiro & Nitschke, 2024).

7. For PubMed searches using the search term “complainant gender,” this phrase was not found in their phrase index. As recommended by PubMed, a proximity search with the term “complainant gender” was instead undertaken with a distance of zero between search items.

8. While Google Scholar contains a moderate amount of gray literature, research suggests that it should not be used alone to conduct gray literature searches. Web of Science was chosen to supplement Google Scholar searches as research suggests that it overlaps very little with Google Scholar (0.2–19.8%) (Haddaway et al., 2015).
